# Developing the Parent-Coaching Assessment, Individualization, and Response to Stressors (PAIRS) Tool for Behavior Analysts

**DOI:** 10.1007/s10803-022-05637-5

**Published:** 2022-06-29

**Authors:** Cressida Pacia, Ciara Gunning, Aoife McTiernan, Jennifer Holloway

**Affiliations:** School of Psychology, National University of Ireland Galway, Galway, Ireland

**Keywords:** Autism, Family-mediated interventions, Parent training, Engagement

## Abstract

Parent engagement in early behavioral intervention is essential to achieving meaningful intervention outcomes. However, parents may experience multiple barriers to engagement. The Parent-coaching Assessment, Individualization, and Response to Stressors (PAIRS) was developed to help practitioners assess families’ barriers and facilitators, individualize their intervention, and respond to stressors using a contextual, functional approach. An expert panel of Board Certified Behavior Analysts ® (BCBAs) evaluated the content validity of the PAIRS. Average scale values (S-CVI/Ave) were 0.92 for relevance, 0.85 for effectiveness, and 0.91 for appropriateness. The PAIRS was revised, and a follow-up evaluation was conducted to rate the tool’s utility. This led to the final version of the PAIRS. Clinical implications and future directions are discussed.

## Introduction

Parent-implemented intervention (PII) is an established evidence-based intervention for improving social communication skills and decreasing problem behavior in children with autism (National Autism Centre, 2015; Steinbrenner et al., 2020), and parent involvement is a critically important element of early behavioral intervention (Division for Early Childhood, 2014; Hyman et al., 2020; National Research Council, 2001). Several systematic reviews (e.g., Akamoglu & Meadan, 2018; Black & Therrien, 2018; Pacia et al., 2021) and meta-analyses (Hampton et al., 2016; Nevill et al., 2018; Roberts et al., 2019) of PIIs have shown positive outcomes in the child’s language and communication, social engagement, and cognitive and adaptive functioning. Additionally, parents who engage in parent training programs have reported improvements in stress (e.g., Dillenburger et al., 2004; Rosenthal et al., 2019), mental health (Tonge et al., 2006), optimism (Koegel et al., 1982), self-efficacy (Iadarola et al., 2018), and parent–child interactions (Koegel et al., 1996; Oono et al., 2013).

Parent engagement is critical for the effectiveness of PIIs for young children with autism (e.g., Oono et al., 2013; Schreibman et al., 2015; Stahmer et al., 2011). Parent engagement has been operationalized in three primary domains: attendance, adherence, and cognitions (Becker et al., 2015; Staudt, 2007). Attendance (i.e., enrollment, session attendance, and completion of treatment) is necessary for the other two components of engagement. Adherence (i.e., participation during sessions and use of strategies between sessions) helps to enhance learning of new skills (Kaminski et al., 2008; Nock & Ferriter, 2005) and increases the likelihood that they are generalized to the family’s everyday life (Karver et al., 2006; Kazantzis et al., 2010). The final component of engagement involves private events such as agreement with treatment rationale, therapeutic alliance, expectations about treatment outcomes, and satisfaction with treatment.

Simply put, engagement involves a parent who enrolls in and attends coaching sessions, applies strategies learned in those sessions to their daily life, and whose needs, preferences, beliefs, and values are in *concordance* with the rationale, goals, and methods of intervention. The term “concordance” has been advanced in related fields to replace the term “adherence” (e.g., Bissonnette, 2008; Dickinson et al., 1999; Snowden et al., 2013). Concordance emphasizes the collaborative relationship between practitioners and parents, such that the practitioner is responsible for ensuring goodness-of-fit between their intervention and the family’s context. The present paper will use the term “adherence” to remain consistent with the parent engagement literature, however, references to concordance will be made where applicable.

### A Functional, Contextual Approach to Engagement

Parent engagement has been identified as the lowest in behavioral interventions, compared with speech-language therapy, occupational therapy, developmental treatments, dietary interventions, and medication (Hock, Kinsman, et al., 2015; Moore & Symons, 2009; Shepherd et al., 2018). Additionally, parents have demonstrated difficulties with using behavioral strategies outside the intervention setting and after intervention has ended (Moore & Symons, 2009, 2011; Pickles et al., 2016). While this may be discouraging to behavioral practitioners, it is important to identify the function – the *why* – of low engagement before attempting to address it.

Allen and Warzak (2000) proposed a functional assessment of parental adherence by presenting an analysis of contingencies, including establishing operations (e.g., failure to establish early outcomes as reinforcers for engagement), stimulus generalization (e.g., insufficient training across different contexts), response acquisition (e.g., excessive complexity of intervention strategies), and consequent events (e.g., competing environmental contingencies). For instance, a parent’s engagement may decline when the intervention strategies fail to produce immediate changes in their child’s behavior. By setting realistic expectations from the outset, the practitioner can establish intermediate outcomes (e.g., child appropriately requesting ice cream instead of hitting) as reinforcers for adherence when access to the parents’ end goal (e.g., child tolerating being denied ice cream) is delayed.

Meanwhile, Fryling (2014) outlined a contextual approach to non-adherence, with an emphasis on addressing their wider context, i.e., potential background factors such as stress or isolation. When practitioners respond to non-adherence without considering the family context, they may inadvertently worsen adherence. For instance, in cases where the function of non-adherence is avoiding stress, simply providing additional training as a blanket response could make adherence even more aversive. By providing a function-based response that takes contextual setting factors into account, such as facilitating breaks or respite, improvements can be made not only in adherence but in the family’s health, well-being, and relationships. Thus, identifying the function of non-adherence and specific barriers to engagement can have a positive impact on intervention outcomes, along with collateral benefits for the family.

### Factors Affecting Engagement

Identifying and functionally addressing barriers to parent engagement is a challenging and prevalent facet of applied practice (Ingersoll et al., 2020), and as such, has garnered research attention, a sample of which is outlined below. The Barriers-to-Treatment Participation Scale (BTPS; Kazdin et al., 1997), which was developed to measure barriers to participation in outpatient therapy for children and families, suggests that barriers to treatment include stressors and obstacles that complete with treatment, treatment demands and issues, perceived relevance of treatment, and relationship with the therapist. While this measure was not specifically intended for families of children with autism, parents of children with autism have indeed been found to be more likely to engage in behavioral interventions if the intervention is perceived to be effective (Bowker et al., 2011; Moore & Symons, 2011; Solish & Perry, 2008) and if it is not too burdensome (Carlon et al., 2013) or complex (Osborne et al., 2008). Other factors that have been found to impact engagement include access to funding, treatment contextualization (i.e., the degree to which the treatment focus matches family needs and the approach fits with their available resources and caregiving style), spousal agreement on goals and strategies, parental confidence and self-efficacy, and perceived acceptance of the child in the family and community (McConnell et al., 2015; Moore & Symons, 2011; Shepherd et al., 2018). Barriers to engagement can be thus multiple and complex, and span a wide range of factors, including logistical, sibling, child, parent, and intervention factors.

Qualitative studies have also been used to elucidate common themes in parents’ barriers and facilitators to treatment engagement (e.g., Amsbary et al., 2020; Hock, Yingling, et al., 2015; Mackintosh et al., 2012; Raulston et al., 2019; Stahmer et al., 2017). The most common themes were parents’ relationship with the practitioner and program logistics such as distance, scheduling, and childcare. Other themes included perceived effectiveness and relevance of the intervention, costs in terms of money, time, and energy, parent stress and overwhelm, social support, effective training approaches, match between intervention design and child skills, and overall satisfaction, particularly relating to improved parent–child relationships. Given the body of literature regarding barriers to engagement, identifying the resources necessary to adhere to the treatment, monitoring the ongoing burden on families, and discussing and working collaboratively to find solutions to anticipated barriers could help improve engagement (Hock, Yingling, et al., 2015).

Variations in the use of PIIs may reflect a mismatch between the interventions and the needs, values, and preferences of families, particularly across socio-economic and cultural groups (e.g., Burkett et al., 2015; Iland et al., 2012; Jegatheesan et al., 2010). Qualitative studies of minority experiences showed unique barriers such as language difficulties, limited services in rural communities, friends and family being dismissive or trivializing, stigma, blaming poor parenting, cultural concerns, and unmet needs from service providers (DuBay et al., 2018; Stahmer et al., 2019). Suggested adaptations and strategies to improve engagement include reduced intervention complexity, improved parent-professional collaboration, more explicit use of family-centered goals, better adult learning strategies, and more peer support for parents (Pellecchia et al., 2018; Stahmer & Pellecchia, 2015).

### Challenges for Behavior Analysts

Despite the importance and the challenges of parent engagement in PIIs, only 27% of behavior analysts report having taken a course on parent training towards their degree or certification, and only 15% reported receiving training in a specific parent training approach (Ingersoll et al., 2020). Behavior analysts reported challenges such as having difficulty engaging families, families not progressing, not having enough skills or knowledge about parent training, and difficulty tailoring parent training to individual families (Ehrhart et al., 2014; Ingersoll et al., 2020; Shapiro et al., 2012).

An important component of parent engagement is therapeutic alliance. Parents of children with autism have reported that a positive and supportive relationship with practitioners contributes to family satisfaction and intervention engagement (e.g., Amsbary et al., 2020; Freuler et al., 2014; Hock, Yingling, et al., 2015). A survey of parents regarding their impressions of behavior analysts’ relationship and compassionate care skills indicated that behavior analysts show some core deficits, such as compromising, inquiring about satisfaction, and not being too authoritarian (Taylor et al., 2018). A follow-up survey by LeBlanc and colleagues (2020) found that most behavior analysts view skills in compassionate care as very or extremely important but received little or no training in these areas. Given the gaps in training and the difficulties behavior analysts have reported in their delivery of parent coaching, practical guidance can help practitioners develop and implement a successful PII.

### A Potential Solution

One potential avenue to assist behavioral practitioners is to provide them with a practical, systematic tool to complement their PII. For example, the BTPS (Kazdin et al., 1997) was developed to measure potential barriers to treatment on a 5-point scale. This scale helps researchers and practitioners identify potential barriers but does not provide guidance in how to address them. Another tool, the Parent and Caregiver Active Participation Toolkit (PACT; Haine-Schlagel & Bustos, 2013), focuses on teaching evidence-based strategies to build a positive therapeutic relationship and increase engagement, such as focusing on strengths and effort and jointly identifying and problem-solving barriers. This toolkit effectively provides guidance on general engagement strategies, but does not focus on identifying unique family factors that impact engagement. A gap persists for a tool that helps practitioners identify barriers and facilitators to engagement, while also providing strategies to improve engagement by systematically adapting a PII to fit an individual family’s needs.

Thus, the purpose of the current study is to develop the Parent-coaching Assessment, Individualization, and Response to Stressors (PAIRS), a tool to help behavioral practitioners identify and address barriers and facilitators to intervention engagement using a contextual, functional approach within a compassionate care framework. The current study sought to develop the PAIRS in three stages and answer the following research questions:

Stage 1: What are potential solutions to common barriers to parent engagement?

Stage 2: Are the solutions to these barriers relevant, effective, and appropriate, as rated by a panel of BCBAs?

Stage 3: Do BCBAs think the PAIRS tool would be useful for implementing PIIs?

## Method

**Fig. 1:**
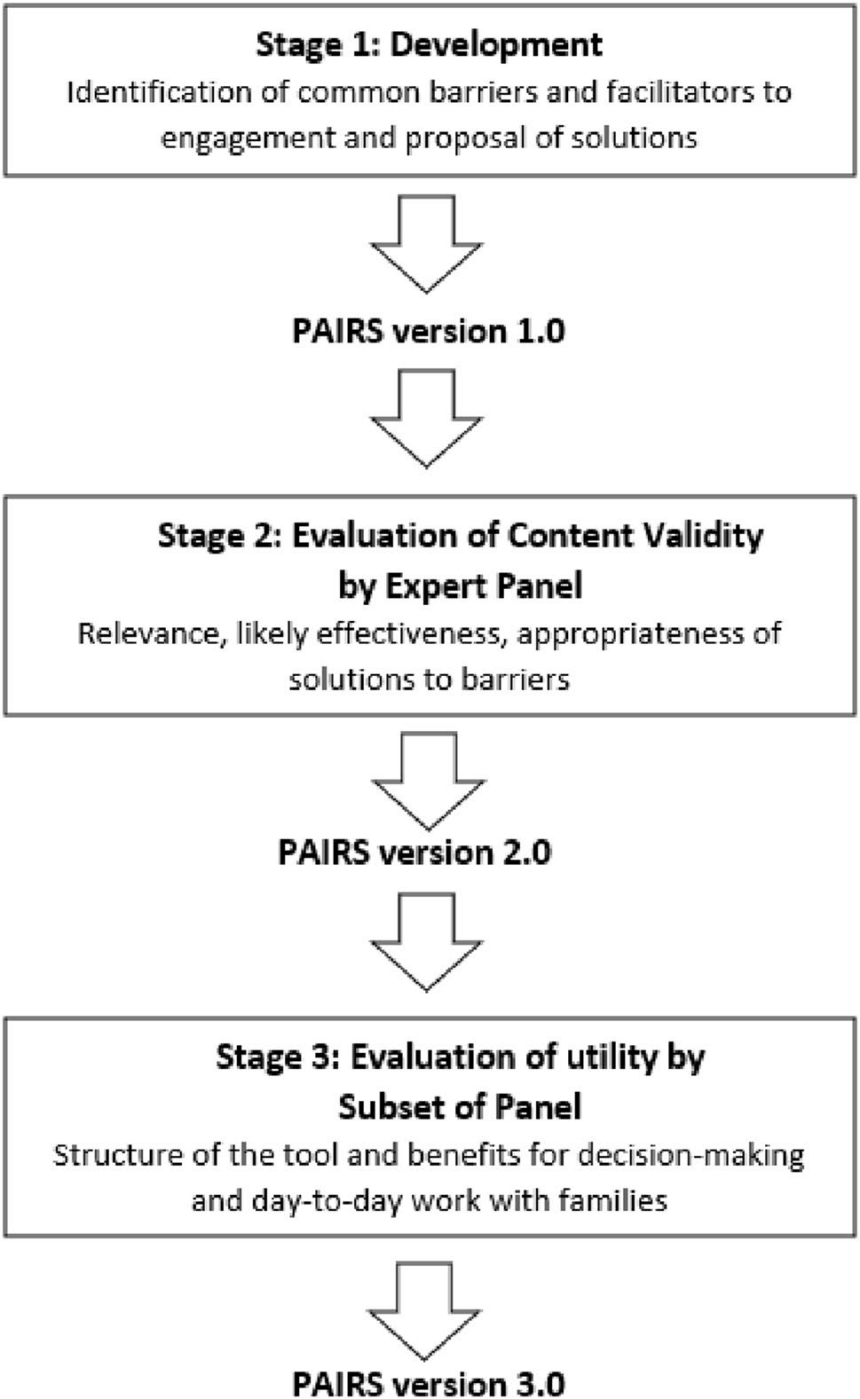
Stages of the development and evaluation of the PAIRS. Adapted from Halek et al., 2017

The process of developing the PAIRS was adapted from the approaches outlined by Kassam-Adams and colleagues (2015) and Halek and colleagues (2017), who developed an e-health intervention for teaching coping skills and an assessment system for challenging behavior in residents with dementia, respectively. To the authors’ knowledge, this approach to tool development is novel in the field of behavior analysis. The development of the PAIRS consisted of three stages (Fig. 1): Stage 1 consisted of a non-systematic collation of barriers and facilitators from the literature and the proposal of function-based solutions to common barriers; Stage 2 consisted of an evaluation by an expert panel of the content validity of the proposed solutions, i.e., relevance of solution to barrier, likely effectiveness of solution to address barrier, and appropriateness for BCBAs to implement solution; and Stage 3 consisted of a follow-up evaluation by a smaller subset of the original panel on the structure of the tool and its potential utility for decision-making and day-to-day work with families. The first evaluation of the PAIRS focused on barrier-solution pairings at the item level using a content validity survey, while the second evaluation examined the tool as a whole by presenting a workshop on the PAIRS and then engaging in discussion and completing a questionnaire.

Parents were not consulted during this early stage of tool development because (1) the barriers, facilitators, and potential solutions were drawn from studies that showcased parent perspectives, allowing the research team to draw from parent experiences to develop the tool without recruiting new parent participants; (2) parent evaluation of the tool is likely to be most valuable and representative when recruited at a later stage of tool development, during real-world clinical practice when they are engaged in a PII; and (3) participation in a later stage of tool development allows the research team to ensure parent participants receive an improved version of the tool, after it has been refined through multiple evaluations.

### Stage 1: Development of the PAIRS

First, common barriers and facilitators were listed, using information obtained from a collation of relevant research. This was not a systematic review of the literature on this topic; the first author identified relevant articles by searching databases using combinations of terms such as “barriers”, “facilitators”, “autism”, “behavior”, “intervention”, and “parents”, and recorded the barriers and facilitators reported. Searches concluded when no new barriers and facilitators were identified through additional searches. Findings from over sixty relevant articles informed the initial list of barriers and facilitators (see Table 1 for a sample). This list was condensed by grouping similar facilitators and barriers together, and then further collapsed by the second author. This was done to allow for enough differentiation to propose distinct function-based solutions, while avoiding an unnecessarily lengthy list of barriers that could impact the practicality and utility of the tool for everyday clinical use.

**Table 1:** Definitions of facilitators and barriers and examples of research

Factor	Definition	Examples of studies drawn from	Study design	Participants
**Facilitators**				
Effective intervention	Parent and child gain knowledge and skills, and family interactions improve. Effective adult learning strategies are used	Bowker et al. (2011) Carlon et al. (2013)	Survey Systematic review	970 parents 2141 parents across 16 studies
Parent self-efficacy and empowerment	Parents are empowered to understand their child’s development and how they could facilitate their child’s developmental successes	Brookman-Frazee (2004) Russell and Ingersoll (2021)	Repeated reversal design Mixed method	3 children and parents 51 parents
Family, peer, and community support	Spousal agreement, peer support, and support and acceptance from extended family and wider community	DuBay et al. (2018) Moore and Symons (2011)	Mixed method Survey	55 parents 21 parents
Child- and family-centred goals and strategies	Contextualization and compatibility with values and lifestyle. Intervention is specific to the needs of the child and provided in a flexible, responsive manner	Coogle and Hanline (2016) Stanford et al. (2020)	Interviews, observations and document analyses Thematic analysis	5 families and 5 service providers 17 mothers
Positive relationship with practitioner	Practitioner uses a non-directive collaborative approach and orients to family strengths	Amsbary et al. (2020) Freuler et al. (2014)	Exploratory qualitative approach Thematic analysis	6 parents 14 families
Additional supports and key provider partnerships	Additional supports are provided where necessary. Collaboration between different service providers is positive and coordinated	Carr and Lord (2016) Helkkula et al. (2020)	Mixed method Qualitative content analysis	8 mother–child dyads 26 parents and 14 service providers
**Barriers**				
*Logistical Factors*				
Difficulties with access	Affordability, availability, and scheduling	Parker and Childs (2019) Pickard and Ingersoll (2016)	Interpretive phenomenological analysis Mixed method	5 parents 244 parents
Administrative difficulties	Difficulties with therapists, equipment, or materials	Grindle et al. (2009) Mackintosh et al. (2012)	Content analysis Web-based qualitative study	53 parents 486 parents
*Child Factors*				
Complex child profile	Child factors that may impact their ability to benefit from intervention such as verbal skills and problem behaviour	Carr et al. (2016) Stahmer et al. (2017)	Randomized controlled trial Mixed method	147 child-caregiver dyads 13 parents
*Sibling Factors*				
Difficulties for siblings	Challenges for siblings such as lack of attention, having to stay out of the way during sessions, or exhibiting increased problem behaviour	McPhilemy and Dillenburger (2013) Pickard et al. (2017)	Survey Mixed method	15 families 103 parents
*Parent factors*				
Individual or cultural concerns	Mismatch between the intervention and individual needs, values, and preferences of families, including cultural factors	Stahmer et al. (2019) Mytton et al. (2014)	Thematic analysis (focus groups and interviews) Systematic review and framework synthesis	58 caregivers and 55 service providers Parents, researchers, and service providers across 26 studies
Difficult circumstances	Circumstances that are challenging for the family, including chronic and/or acute stressors	Hock, Yingling, et al. (2015) Wicks et al. (2019)	Qualitative analysis Hierarchical multiple regression analyses	13 parents 97 mothers
Treatment burden	Demands placed on the child and family during intervention are too high	Kazdin et al. (1997) Rivard et al. (2020)	Prospective study Content analysis	260 families 28 parents
Training not the right fit	Mismatch between parent needs/preferences and intervention characteristics such as training model, adult learning strategies, or treatment complexity	Leadbitter et al. (2020) Raulston et al. (2019)	Thematic analysis Iterative content analysis (focus groups)	18 parents 30 parents
Low motivation or belief in effectiveness	Low or fluctuating motivation to engage in intervention and/or low belief that the intervention will be effective	Shepherd et al. (2018) Solish and Perry (2008)	Survey Survey	570 parents 48 parents and 34 therapists
*Intervention factors*				
Variations in treatment efficacy	Child not progressing as expected, slow progress, or regression in target skills	Botterill et al. (2019) Manohar et al. (2020)	Thematic analysis Thematic content analysis	8 parents 30 families
Difficulties with generalization	Difficulties with generalization or maintenance of skills learned in intervention	Mitteer et al. (2018) Strauss et al. (2012)	Laboratory model Hierarchical linear regression analyses	4 caregivers 44 families

Specific barrier-solution pairings were then delineated by proposing potential function-based solutions to address each identified barrier. Solutions were developed based on findings drawn from the literature, including identified facilitators to engagement (see Table 1 for a list of common facilitators) and specific recommendations provided by parents when discussing their barriers and experiences with PIIs (e.g., Amsbary et al., 2020; Stahmer et al., 2011). Solutions were also informed by the collective professional experience of the research team, which comprises two BCBAs and two BCBA-Ds. This led to the first version of the PAIRS (version 1.0), which comprised a table of barriers and potential solutions.

### Stage 2: Evaluation of Content Validity

This stage of evaluation focused on the content validity (i.e., relevance, likely effectiveness, and appropriateness) of each item (barrier-solution pairing). The purpose of this stage was to investigate which solutions needed to be revised or discarded, and to obtain suggestions from an expert panel on additional function-based solutions to address each barrier. This stage of tool development focused on recruiting feedback from BCBAs as the intended administrators of the tool. The research team intends to gain feedback from parents in future studies through the use of the tool in real-world settings, after the tool has been refined through the evaluations and revisions presented here.

#### Participants and Recruitment

Twenty-eight BCBAs were invited to participate, with a response and completion rate of 53.6% (15 participants). This is consistent with recommendations for the first iteration of content validation, which suggests a panel of 8–12 experts (Lynn, 1986; Polit et al., 2007). The research team recruited BCBAs who work regularly with parents and caretakers by sending an email invitation to participate in an expert panel (i.e., convenience sampling). Eligibility requirements for participation included certification with the Behavior Analyst Certification Board (BACB) and professional experience delivering parent training interventions. BCBAs were recruited from several countries, including the United Kingdom, Ireland, Australia, and the United Arab Emirates. The majority of participants reported 11–20 years of experience in behavior analysis and served clients with ASD aged 3–12 (see Table 2 for expert panel demographics). Only responses from BCBAs who completed the survey in full were included in the analysis.

**Table 2:** Demographics of expert panel

Category	Demographics	Number	Proportion
Gender	Male	3	20%
	Female	12	80%
Certification	BCBA	13	86.7%
	BCBA-D	2	13.3%
Highest level of education	MA/MSc	12	80%
	PhD	3	20%
Years of experience	6–10 years	4	26.7%
	11–20 years	10	66.7%
	Over 20 years	1	6.7%
Area of emphasis^a^	Early Intensive Behavioural Intervention (EIBI)	13	86.7%
	Positive Behaviour Support (PBS)	10	66.7%
	Activities of Daily Living (ADLs)	13	86.7%
	Social and Communication skills	13	86.7%
	Sleep issues	4	26.7%
	Feeding issues	6	40%
	Academic/Educational skills	7	46.7%
	Vocational/Employment skills	3	20%
	Other^b^	1	6.7%
Types of parent training provided^a^	In-person	15	100%
	Telehealth	7	46.7%
	Individual	11	73.3%
	Group	6	40%
	Primary (all parent-mediated)	10	66.7%
	Secondary (therapist-delivered with some PT)	11	73.3%
	Sibling training	1	6.7%
	Other	1	6.7%
Typical length of parent training services	4–12 weeks	2	13.3%
	Over 12 months	6	40%
	Variable/consultative basis	7	46.7%
Age of clients^a^	0–2	7	46.7%
	3–6	14	93.3%
	7–12	11	73.3%
	13–17	10	66.7%
	18–25	7	46.7%
	26–40	4	26.7%
	41–64	1	6.7%
	65 and above	1	6.7%
Typical client profile^a^	Autism Spectrum Disorder	15	100%
	Intellectual/Developmental Disabilities	11	73.3%
	Emotional or Behavioural Disorders	3	20%
	Typically Developing	3	20%
Approximate number of families who have received PT services	10 or fewer	2	13.3%
	11–20	3	20%
	21–30	4	26.7%
	31–50	5	33.3%
	Over 50	1	6.7%

^a^Participants can choose more than one option

^b^Human Rights and Challenging Behaviour (restrictive practices, restraint reduction)

#### Instrument and Distribution

The Content Validity Survey Tool (CVST; Kassam-Adams et al., 2015) was adapted for the current study and the three dimensions of content validity were revised to apply to barriers and proposed solutions: (1) relevance, i.e., the extent to which a solution is pertinent to its barrier; (2) likely effectiveness, i.e., the extent to which the solution would successfully address its barrier; and (3) appropriateness of the solution for BCBAs delivering parent training services. The proposed solutions from PAIRS version 1.0 were divided into two groups: intervention adaptations and additional services or referrals. This was done to streamline the content validity evaluation by only presenting the intervention adaptations in the CVST. The adapted CVST was thus populated with 45 barrier-solution pairings, consisting of eleven barriers and 2–6 proposed solutions per barrier. Response options were on a four-point scale ranging from “not at all” to “very”. The CVST also contained a suggestion box for reviewer comments after each barrier and a final section for comments on the PAIRS as a whole. The CVST was uploaded to Qualtrics, an online survey tool, and a link to the survey was emailed to all invited BCBAs.

#### Analysis

Results of the CVST were analyzed quantitatively (via calculation of content validity indices; CVI) and qualitatively (via examination of narrative comments). The CVI was calculated for each scale of item (I-CVI), i.e., the relevance, effectiveness, and appropriateness of each barrier-solution pairing, and each scale overall (S-CVI/Ave), i.e., the solutions’ overall relevance, effectiveness, and appropriateness. For each item, the I-CVI was computed as the number of experts giving a rating of either 3 or 4 divided by the number of experts. The recommended minimum I-CVI value for five or more experts is 0.78 (Polit et al., 2007). The average agreement for each scale overall (S-CVI/Ave) was calculated by calculating the average I-CVI across all items. The recommended minimum value for S-CVI/Ave is 0.9 (Polit et al., 2007). Qualitative comments were categorized into suggestions and comments, and the suggestions were grouped into similar factors within each barrier to allow for narrative examination. The PAIRS table of barriers and solutions was then refined based on the combined quantitative and qualitative findings. To better support practitioners for whom the PAIRS is intended, a more comprehensive tool was developed with additional components derived from this table that corresponded with the typical stages of implementing a PII (i.e., developing and planning, conducting an intake assessment, and finally implementing the intervention). This expanded tool was labelled PAIRS version 2.0.

### Stage 3: Evaluation of Utility

While the first iteration of evaluation focused on barrier-solution pairings at the item level, this second iteration sought feedback on the revised tool as a whole, specifically its structural aspects and potential benefits for decision-making and day-to-day work with families. This was done through a workshop presenting PAIRS version 2.0 to a subset of the original panel, followed by a discussion and questionnaire.

#### Participants and Recruitment

In line with previous recommendations, a subset of the same experts who participated in the primary evaluation of an instrument re-evaluated the revised version (Halek et al., 2017; Lynn, 1986). All the members of the original panel were invited via email to attend the PAIRS workshop and re-evaluation (*n* = 15). Six members of the panel were unable to attend due to scheduling conflicts, two members registered for the workshop but did not attend, and three members did not respond to the invitation. Four members of the panel attended the workshop and participated in the re-evaluation. Caution should be taken when evaluating the results of the second evaluation due to the relatively small number of participants, although the size of the panel is consistent with the recommendation of recruiting 3–5 reviewers for a secondary review (Polit et al., 2007).

#### Workshop

The workshop took place over Zoom, an online meeting platform. The first author delivered the one-hour workshop to the subpanel. At the beginning of the workshop, participants were asked to describe a family they had worked with who they found difficult to engage, and the circumstances that made it challenging. Participants were then asked to keep this family in mind as the PAIRS was introduced, and to consider how having access to the PAIRS may have impacted how they worked with the family. An introduction to the background and rationale of the PAIRS was presented, including the functional, contextual approach to engagement and the role of concordance. This was followed by an overview of the results from the first evaluation (i.e., CVI, narrative comments, and item revisions), which they had participated in. Finally, the PAIRS tool was presented and explained in full.

#### Evaluation and Analysis

At the end of the workshop, participants engaged in a brief discussion and were then provided a link to a questionnaire. The questionnaire had four sections: (1) Structure; (2) Benefits of the tool for planning and decision-making; (3) Benefits of the tool for day-to-day work with families; and (4) Overall impression of the tool. Response options were on a four-point scale ranging from “strongly disagree” to “strongly agree.” Comments and suggestions were requested at the end of each section. The discussion portion of the workshop was also transcribed and qualitatively examined. Questionnaire data (quantitative and qualitative) were reviewed. The PAIRS was then further revised based on this feedback (PAIRS version 3.0).

## Results

Stage 1 results included the collation of common barriers and facilitators and the development of potential solutions, Stage 2 results included the quantitative (i.e., content validity indices) and qualitative (i.e., examination of narrative comments) findings from the content validity evaluation, and Stage 3 results included descriptive statistics from the questionnaire and revisions based on the post-workshop discussion and written comments.

### Stage 1: Development

Forty-eight barriers and 41 facilitators were identified. After grouping similar factors together, 11 barriers and six facilitators remained (Table 1). Multiple solutions were proposed for each barrier (Table 3). For example, for the barrier “Difficulties with access”, four solutions were proposed, including connecting families with charities, insurance, funding supports, etc., where appropriate, providing Telehealth or home-based training, providing flexible training days/times, and developing explicit strategies for involving the child and/or sibling. Thus, PAIRS Version 1.0 consisted of a list of 6 facilitators and a table of barriers and proposed solutions. This table comprised 11 barriers and 2–6 solutions per barrier, for a total of 45 barrier-solution pairings.

**Table 3:** Barriers with original and revised solutions based on I-CVI values and narrative suggestions

Barrier	Original solutions	I-CVI values			Relevant comments/suggestions	Revised solutions
		Relevance	Effectiveness	Appropriateness		
Difficulties with access	Connect families with charities, insurance, funding supports, etc., where appropriate	0.87*	0.73	0.8*	Some comments noted how these were not possible for them	Connect families with charities, insurance, funding supports, etc., where possible and appropriate
	Telehealth or home-based training	1*	0.93*	0.93*	Include pre-recorded trainings	Telehealth, self-directed learning, or home-based training
	Flexible training days/times	0.93*	0.87*	0.93*	Flexibility to follow up with staff and consultants on a regular basis when issues arise; Re-scheduling	Flexible training days/times, including flexibility to reschedule or follow up with staff as needed
	Explicit strategies for involving child/sibling (e.g., sibling training component)	0.73	0.67	0.87*	Approach family as a whole early on	Approach family as a whole, including strategies for occupying or involving child and siblings
	N/A	N/A	N/A	N/A	Coordinated team approach to scheduling of appointments	Coordinate a team approach to scheduling of appointments
	N/A	N/A	N/A	N/A	Providing childcare, meals or meeting other needs for families	Provide or connect family with childcare, meals, or other needs
Administrative difficulties	Telehealth or clinic-based training	0.73	0.73	0.8*	Easy to access modes of contact, e.g., phone, WhatsApp, Teams	Flexible models of service delivery, including easy-to-access modes of contact (e.g., WhatsApp)
	Provide (or reduce need for) additional therapy materials	0.87*	0.87*	0.67	Online portal to request materials; Toy/game lending library; Use of apps and software	Provide (or reduce need for) additional therapy materials (e.g., apps and software, online portal to request materials, toy lending library)
	Collaboratively problem-solve on setting up home environment or choosing appropriate community settings	0.87*	0.87*	0.8*	N/A	*Keep same solution*
	N/A	N/A	N/A	N/A	Provide parent-friendly data sheets	Provide parent-friendly data sheets
Complex child profile	Individualize child programming (e.g., adaptations for non-verbal children)	1*	1*	1*	N/A	*Keep same solution*
	Multidisciplinary collaboration (e.g., with SLT, OT)	0.93*	0.73	0.93*	MDT should meet to discuss recommendations and use an agreed framework to decide order of recommendations and how to evaluate effectiveness	Multidisciplinary collaboration (e.g., with SLT, OT) using a coordinated, positive approach
	Additional parent training in Positive Behaviour Support	1*	1*	1*	Provide an understanding of the function of behaviour and a collaboratively designed treatment plan; Parents need a basic understanding of PBS prior to seeing you	Additional parent training in Positive Behaviour Support, including a basic understanding of PBS prior to intervention and clear mutual understanding of the function of behaviour
Difficulties for siblings	Identify and collaboratively problem-solve source of sibling difficulty	1*	0.93*	0.93*	N/A	*Keep same solution*
	Explicit strategies for including siblings (e.g., sibling training, parent training specific to sibling)	1*	0.8*	0.87*	Sibling support group, e.g., Sibshops	Explicit strategies for including siblings (e.g., sibling training, sibling groups)
Individual or cultural concerns	Clarify values and goals at the outset and on an ongoing basis	1*	0.8*	1*	N/A	*Keep same solution*
	Collaborate with both parents on goal-setting, selecting target behaviour, and implementation strategies	1*	0.87*	0.93*	N/A	*Keep same solution*
	Provide clear rationale for intervention strategies	0.93*	1*	0.93*	N/A	*Keep same solution*
	Shape successive approximations and establish intermediate outcomes as reinforcers (e.g., when trying to transition away from discipline practices such as spanking)	0.67	0.73	0.67	Several comments focused on the example of spanking so example was changed	Shape successive approximations and establish intermediate outcomes as reinforcers (e.g., when trying to transition away from discipline practices such as yelling)
	Collaborate with (or refer to) professionals from the same culture where appropriate	0.8*	0.87*	0.8*	Needs of second-language learners	Collaborate with (or refer to) professionals from the same culture where appropriate, especially if there is a language barrier
	Seek training in cultural competence (e.g., on maintenance of cultural identities, upholding religious practices, role of extended family and community, gender roles)	0.93*	0.93*	0.93*	N/A	*Keep same solution*
Difficult living circumstances	Multidisciplinary collaboration (e.g., with social worker)	0.93*	0.87*	0.93*	N/A	*Keep same solution*
	Reduce treatment burden and/or complexity	1*	0.73	1*	N/A	*Keep same solution*
	Increase treatment relevance	0.93*	0.93*	0.93*	N/A	*Keep same solution*
	Collaborative problem-solving (e.g., around structural barriers)	1*	0.93*	0.93*	N/A	*Keep same solution*
	N/A	N/A	N/A	N/A	Outreach/respite; Identify whether other services need to be prioritized	Connect with outreach/respite services, which may need to be prioritized over behavioural services
Treatment burden and stress	Identify source of stress (e.g., demand for involvement too high, lack of time for other activities)	1*	0.8*	0.93*	N/A	*Keep same solution*
	Reduce treatment burden, intensity, and/or complexity	1*	0.93*	1*	N/A	*Keep same solution*
	Regular check-ins with family	0.93*	0.87*	0.93*	N/A	*Keep same solution*
	Flexible training days/times	0.87*	0.8*	0.93*	Mixed models e.g., in person and online	Flexible training days/times and models of delivery (e.g., blended models)
	Distance-learning options	0.8*	0.6	0.87*	N/A	*Incorporated into above solution*
	Proactively problem-solve for challenging situations	0.93*	0.93*	0.93*	N/A	*Keep same solution*
	N/A	N/A	N/A	N/A	Incorporate ACT; Identify stage of readiness of change	Incorporate ACT strategies
	N/A	N/A	N/A	N/A	Outreach/respite	Connect family with outreach/respite services
Training not the right fit	Provide different training options (e.g., self-directed, Telehealth, individual, group, etc.) depending on parent needs/preferences	0.93*	0.73	0.8*	N/A	*Keep same solution*
	Teaching materials incorporate a variety of effective adult learning strategies	0.93*	0.87*	1*	N/A	*Keep same solution*
	Adjust intervention complexity as needed	0.87*	0.87*	0.93*	N/A	*Keep same solution*
	N/A	N/A	N/A	N/A	Incorporate ACT	Incorporate ACT strategies
Low motivation/belief in effectiveness	Collaborate with both parents on goal setting, selecting target behaviour, and implementation strategies	0.87*	0.87*	0.93*	N/A	*Keep same solution*
	Establish intermediate outcomes as reinforcers	1*	0.93*	1*	N/A	*Keep same solution*
	Establish treatment relevance through regular check-ins on priority family goals	0.87*	1*	0.93*	N/A	*Keep same solution*
	Share parent-friendly data sheets	0.8*	0.67	0.87*	N/A	Share parent-friendly graphs to show progress
	Create behavioural momentum by setting smaller, achievable goals at the start and as needed throughout the intervention	1*	1*	1*	N/A	*Keep same solution*
	N/A	N/A	N/A	N/A	Incorporate ACT	Incorporate ACT strategies
	N/A	N/A	N/A	N/A	Detailed discussion of previous interventions and the reasons for why those interventions were not effective	Discuss previous interventions in detail and why they were not effective
	N/A	N/A	N/A	N/A	N/A	*Moved from barrier “Difficulties with Generalization”:* Connect with peer support (e.g., other parents who completed the same training)
Variations in treatment efficacy	Explicitly acknowledge that strategies may not always be effective or that there may be setbacks and plan for this early on^a^	0.93*	0.53	1*	Set expectations and highlight level of commitment and involvement; Realistic expectations for how often data is collected, how long they will need to collect it, and a plan for when it's reviewed	Set clear and realistic expectations at the outset, including levels of involvement, a plan for collecting and reviewing data, and explicit acknowledgment that there may be setbacks
	Involve parents in problem-solving solutions and adaptations	1*	0.87*	0.93*	Give general info and then collaboratively problem-solve; Shared understanding of function of behaviour	Promote shared understanding and collaborative problem-solving
	Program for generalization	0.93*	1*	1*	N/A	*Keep same solution*
	Share parent-friendly data sheets	0.93*	0.87*	0.93*	N/A	*Keep same solution*
	N/A	N/A	N/A	N/A	Incorporate ACT	Incorporate ACT strategies
Difficulties with generalization	Program for generalization	1*	1*	1*	N/A	*Keep same solution*
	Discuss and problem-solve potential difficulties in advance (e.g., implementing strategies around disapproving family members)	1*	0.87*	1*	N/A	*Keep same solution*
	Connect with peer support (e.g., other parents who completed the same training)	0.87*	0.73	0.73	N/A	*Moved to Low motivation/belief in effectiveness*
	Incorporate a self-management system	0.93*	0.8*	0.93*	Develop a reinforcer system for the parents for their fidelity to treatment behaviours	Incorporate a self-management system or work with parent to recruit reinforcement (e.g., spouse)
	Establish a check-in schedule (e.g., short check-ins between sessions, monthly check-ins after completion of training)	0.93*	0.87*	0.93*	Tailor amount of contact	Establish an individualized check-in schedule (e.g., short check-ins between sessions, monthly check-ins after completion of training)
Total	S-CVI/Ave	0.92**	0.85	0.91**	N/A	N/A

*I-CVI* Item-Content Validity Index, *S-CVI/Ave* Scale-Content Validity Index/Average Agreement

*Above recommended I-CVI value (0.78)

**Above recommended S-CVI/Ave value (0.9)

### Stage 2: Evaluation of Content Validity

The 45 barrier-solution pairings were reviewed by fifteen BCBAs for content validity (i.e., relevance, effectiveness, and appropriateness) (see Table 3). I-CVI values ranged from 0.53 (*N* = 1) to 1 (*N* = 35), which is the highest possible I-CVI value. The recommended minimum I-CVI value for five or more experts is 0.78 (Polit et al., 2007). Content validity indices across barrier-solution pairings are presented in Table 3. Thirty-three (73.3%) items had I-CVI values at or above the recommended minimum value across all three scales. Eight items (17.8%) had one scale below the recommended value, while four items (8.9%) had two scales below the recommended value. Overall scale values were above the recommended minimum value of 0.9 for relevance (S-CVI/Ave = 0.92) and appropriateness (S-CVI/Ave = 0.91), but slightly below the recommended minimum value for effectiveness (S-CVI/Ave = 0.85).

#### CVI for Logistical Factors

For the barrier “Difficulties with access”, two solutions (50%) had I-CVI values above the recommended value for all scales. The proposed solution to connect families with charities, insurance, funding supports, etc. was below the recommended value for effectiveness (I-CVI = 0.73) and the solution to include explicit strategies for involving child/sibling was below the recommended value for relevance (I-CVI = 0.73) and effectiveness (I-CVI = 0.67). For the barrier “Administrative difficulties”, one solution (33.3%) had I-CVI values above the recommended value across all scales. One solution (Provide or reduce need for additional therapy materials) had was below the recommended value for appropriateness (I-CVI = 0.67), and one solution (Telehealth or clinic-based training) was below the recommended value for relevance (I-CVI = 0.73) and effectiveness (I-CVI = 0.73).

#### CVI for Child and Sibling Factors

For the barrier “Complex child profile”, two solutions (66.7%) had I-CVI values of 1 across all scales, which represents the maximum value. One solution (Multidisciplinary collaboration) was below the recommended value for effectiveness (I-CVI = 0.73). For the barrier “Difficulties with siblings”, both solutions (100%) had I-CVI values above the recommended value across all scales.

#### CVI for Parent Factors

For the barrier “Individual or cultural concerns”, five solutions (83.3%) had I-CVI values above the recommended value across all scales. One solution (Shape successive approximations and establish intermediate outcomes as reinforcers) was below the recommended value for relevance (I-CVI = 0.67) and effectiveness (I-CVI = 0.73). For the barrier “Difficult living circumstances”, three solutions (75%) had I-CVI values above the recommended value across all scales. One solution (Reduce treatment burden and/or complexity) was below the recommended value for effectiveness (I-CVI = 0.73). For the barrier “Treatment burden and stress”, five solutions (83.3%) had I-CVI values above the recommended value across all scales. One solution (Distance-learning options) had was below the recommended value for effectiveness (I-CVI = 0.6). For the barrier “Training not the right fit”, two solutions (66.7%) had I-CVI values above the recommended value across all scales. One solution (Provide different training options) was below the recommended value for effectiveness (I-CVI = 0.73). Finally, for the barrier “Low motivation/belief in effectiveness”, four solutions (80%) had I-CVI values above the recommended value across all scales. One solution (Share parent-friendly data sheets) had was below the recommended value for effectiveness (I-CVI = 0.67).

#### CVI for Intervention Factors

For the barrier “Variations in treatment efficacy”, three solutions (75%) had I-CVI values above the recommended value across all scales. One solution (Explicitly acknowledge that strategies may not always be effective or that there may be setbacks and plan for this early on) was below the recommended value for effectiveness (I-CVI = 0.53). For the barrier “Difficulties with generalization”, four solutions (80%) had I-CVI values above the recommended value across all scales. One solution (Connect with peer support) had was below the recommended value for effectiveness (I-CVI = 0.73) and appropriateness (I-CVI = 0.73).

#### Narrative Suggestions

Narrative suggestions were examined for overarching factors leading to the identification of six common factors: Flexibility and accommodation, coordination between services, addressing additional needs/providing additional services, incorporating Acceptance and Commitment Therapy (ACT), parent-practitioner collaboration, and proactive planning and setting of initial expectations. These factors were consistent with identified facilitators from the literature, except for incorporating ACT and proactive planning and setting of initial expectations. These two suggestions were novel findings and were incorporated into the proposed solutions. The narrative comments were then divided into comments and specific suggestions. For each barrier, similar suggestions were grouped together and summarized for examination. Individual solutions were then revised based on a combination of the quantitative and qualitative feedback (see Table 3 for revised solutions and the relevant comments/suggestions).

#### Revision

The table of barrier-solution pairings from PAIRS Version 1.0 was updated with the revised solutions. To support practitioners using the tool, additional components of the PAIRS were derived (see Table 4), and the table of barrier-solution pairings was named the PAIRS Table of Function-Based Solutions. As using a strengths-based approach was identified as a facilitator, facilitators are always presented before barriers within each component of the tool.

**Table 4:** Comprehensive PAIRS components

Name	When to use	Purpose	Content
Introduction to the PAIRS	First time using the PAIRS As needed for clarification or guidance	Provide background information and instructions on how to use the tool	Information on the purpose and development of the PAIRS An overview of the components of the PAIRS A flowchart of how to use the PAIRS in practice
PAIRS Good Practice Checklist for Practitioners	Developing or planning general PII	Provide practitioners with strategies to develop an intervention that is likely to promote parent engagement, by building on common facilitators and avoiding negative effects of common barriers	Instructions for using the Checklist 23 items mapping onto identified facilitators and barriers Potential adjustments to intervention factors Suggested practitioner skills and behaviors
PAIRS Assessment and Individualization Plan	Intake with a new family As needed when parent engagement is low or decreasing	Aid practitioners in gathering information about a family’s unique barriers and facilitators, identifying the most critical barriers and facilitators, and collaboratively developing a plan to adapt the intervention or provide additional services or referrals	Instructions for using the Assessment and Individualization Plan A sample completed Assessment and Individualization Plan 30 items mapping onto identified facilitators and barriers Space to list top 3 facilitators and top 3 barriers Space to record intervention adaptations and/or additional services and referrals
PAIRS Table of Function-based Solutions	In conjunction with the PAIRS Assessment and Individualization Plan	Suggest potential function-based solutions to common barriers as a guide for collaborative problem-solving with the parent	Instructions for using the Table 50 suggestions for intervention adaptions 9 suggestions for additional services and referrals
Appendix	First time using the PAIRS As needed for clarification or guidance	Provide operational definitions of terms used in the PAIRS and suggestions for further reading	Definitions of 6 facilitators Definitions of 11 barriers List of potentially unfamiliar terms and concepts with suggestions for further reading (e.g., compassionate care, ACT)

### Stage 3: Evaluation of Utility

The four members of the sub-panel responded “agree” or “strongly agree” to all items on the questionnaire, including items evaluating the structure of the PAIRS (i.e., clarity, meaningfulness, completeness, length and scope, and comprehensibility), its potential benefits as for day-to-day work with families (i.e., time required and utility as an information aid, communication aid, planning aid, and evaluation aid), potential benefits as a tool for assessment and decision-making (see Table 5), and overall assessment of the PAIRS (i.e., usefulness and practicability, use in day-to-day work, recommendation to other BCBAs).

**Table 5:** Questionnaire results for PAIRS benefits for day-to-day work with families

Item	Number of ratings^a^	
	Agree	Strongly agree
The PAIRS has encouraged me to think more intensively about the family’s contextual barriers	0	4
The PAIRS contains factors that I had not thought about in the context of the family’s intervention engagement and progress	0	4
I see a relationship between the PAIRS and my daily work	1	3
By using the PAIRS, I see the family’s challenges from another perspective	0	4
With the PAIRS, I can better describe the family’s barriers and facilitators to others (e.g., colleagues, multidisciplinary teams)	0	4
Planning and adapting interventions to address the family’s barriers is easier with the information provided by the PAIRS	1	3
Using the PAIRS provides information about the families that I did not know before	1	3
Since the introduction of the PAIRS, I have a better understanding of the barriers the family faces	2	2
I feel that the family’s engagement and progress will change positively with the introduction of the PAIRS	2	2
The PAIRS gives me the opportunity to build on the strengths and individuality of the child and family	2	2
The PAIRS will help me build and maintain a positive collaborative relationship with the family	2	2
The PAIRS provides helpful guidance for professional development and further reading	2	2

^a^No ratings were received for “Strongly disagree” or “Disagree”

Comments were also received within each of the four sections. Based on a combination of the verbal (during discussion) and written (from questionnaire) comments and suggestions, the following revisions were made: (1) Background and rationale were added to the tool, as a participant noted that concepts introduced during this section of the presentation were impactful; (2) Contents of the appendix (definitions of barriers and facilitators and a list of terms) were moved to the front of the tool to improve clarity; (3) Practitioner skills and behaviors (i.e., using a non-directive collaborative approach, using a strengths-based approach) were operationalized more clearly within the body of the tool, as suggested by a participant; (4) Additional readings were added to the list of terms, including specific suggestions from participants; (5) Motivational Interviewing was added as a potential solution alongside ACT, as suggested by a participant; (6) A section of “Additional Considerations and Limitations” was added with advice on administration, adaptation, and limits of the tool, based on comments from multiple participants during the discussion.

## Discussion

The PAIRS was developed grounded in the functional contextual approach to engagement (Allen & Warzak, 2000; Fryling, 2014; Moore & Amado, 2021) and in response to recent calls for increased attention to compassionate care for behavior analysts (Callahan et al., 2019; Leblanc et al., 2020; Taylor et al., 2018). The PAIRS was designed to collate the information drawn from a growing body of literature examining common barriers and facilitators to engagement and make it accessible to practitioners delivering PIIs, and help them tailor their intervention to individual families. In the first evaluation of the PAIRS by a panel of fifteen BCBAs, the overall average rating of the relevance and appropriateness of all proposed solutions were each over 0.9, indicating that on average, over 90% of ratings agreed that the proposed solutions were relevant to the barriers and appropriate for use by BCBAs. The average rating of the effectiveness of the solutions was slightly lower, at 0.85. This indicates that on average, 85% of ratings agreed that the proposed solutions were likely to be effective at addressing the barriers. One potential explanation for this slightly lower rating for effectiveness is that family barriers are complex and are unlikely to be easily addressed with a single solution. Thus, the individual barrier-solution pairings presented in the CVST may have been rated as less effective at the item level since multiple solutions were proposed for each barrier. Nevertheless, it is worthwhile to note that 85% is still a high level of agreement.

While the first evaluation of the PAIRS focused on evaluating the tool at the item level, the second evaluation examined the tool as a whole. A subset of the original panel attended a workshop which introduced them to the comprehensive PAIRS tool. It should be noted that the second evaluation was conducted with only four participants. While this is in line with recommendations for studies of content validity (e.g., Polit et al., 2007), their evaluation may not fully generalize to the wider behavior analytic community. Nevertheless, in a discussion at the end of the workshop, all participants noted the lack of training and guidance they had received on how to work with parents. One participant noted, “A lot of the things you’ve gone through in the last hour [of the workshop], it just made me think, where have all these things been over the last few years?” This is consistent with Ingersoll and colleagues’ (2020) findings that the majority of behavior analysts have not received adequate support in how to effectively conduct parent training. All participants highly rated the PAIRS’ structure and benefits for decision-making and day-to-day work with families. One participant commented, “I see it becoming part of the essential guides required to implementing successful parent training. Its comprehensive and user-friendly layout is exactly what is needed for behavior analysts to effectively support and train parents.”

The PAIRS was designed to be complementary and collaborative. That is, the PAIRS is intended to complement an evidence-based intervention; it is not a standalone program. The use of a tool to enhance and complement existing interventions is preceded by PACT, a toolkit for promoting parent engagement (Haine-Schlagel & Bustos, 2013). For instance, PACT has been integrated into Project ImPACT (Ingersoll & Dvortcsak, 2010), an evidence-based PII for improving social communication skills in children with ASD. The engagement strategies are incorporated into the parent training lessons, e.g., when teaching parents of children with ASD how to make play interactive, practitioners are encouraged to make suggestions rather than giving directions (Rieth et al., 2018). While PACT provides good-practice strategies that are helpful for practitioner interactions with most families, the PAIRS provides strategies for individualizing interventions based on specific family variables. Thus, the PAIRS can similarly be incorporated into a PII, where it can provide systematic guidance to practitioners on jointly identifying barriers and facilitators and problem-solving challenges unique to each family, consistent with building a positive and empowering therapeutic relationship. Furthermore, the PAIRS is complementary in that it provides information and suggestions for further reading and training for skills and concepts that may be unfamiliar to practicing behavior analysts (e.g., therapeutic relationship skills), but may be required to produce the greatest benefit.

The PAIRS is also intended to be used collaboratively. The function-based solutions presented are intended to guide the collaborative problem-solving process with families, not to prescribe rigid responses to complex situations. The PAIRS thus encourages concordance rather than strict adherence and emphasizes open communication and joint problem-solving. This is supported by the narrative comments received. For example, one reviewer noted “…the importance of including the parents and facilitating them to elicit the solutions for change, as then there is more buy in, and it reduces the ‘expert model’ of the parents relying on you to solve this problem.” Another reviewer commented, “Adopting a collaborative approach, which listens carefully to the concerns of the family, is vital. This is an ongoing ‘soft’ skill for BCBAs to develop and requires ongoing work.” During the second evaluation discussion, several members of the panel noted how going through the PAIRS with a family could be a way to connect and foster concordance by helping practitioners better understand the family’s context.

This is in line with improving relationship and compassionate care skills for behavior analysts. In the second evaluation of the PAIRS, the four members of the secondary panel strongly agreed that the PAIRS encouraged them to think more intensively about the family’s contextual barriers and see the family’s challenges from another perspective. One participant commented, “The categories of barriers and facilitators help me to think through other idiosyncratic variables specific to the family and how they might fit within the PAIRS framework.” By illustrating the contextual variables that commonly impact families of children with ASD and providing guidance on potential areas of professional development, the PAIRS seeks to help practitioners build positive therapeutic alliance with families. The feedback received from the panel is encouraging in this regard, however further evaluation by both practitioners and families in real-world clinical settings is needed.

When considering the barriers and facilitators to engagement within a family system, the PAIRS takes a multi-level approach, with the chosen level of analysis defined by its pragmatic purpose (Hayes et al., 2021). That is, the PAIRS expands the traditional behavioral paradigm from the level of the individual to the level of the family, and from immediate environmental contingencies to nonlinear contextual variables, including private events (e.g., values and beliefs) on one end of the spectrum and large-scale external factors (e.g., socioeconomic disadvantage, cultural concerns) on the other. This breadth of scope is necessary because the barriers and facilitators to engagement identified in the literature cover this continuum. The PAIRS recognizes that a single practitioner cannot address all the potential challenges a family may experience. Thus, the PAIRS encourages practitioners to focus on adaptations that they can make within their program, and advocates for appropriate referrals to other services as well as positive and coordinated multi-disciplinary collaboration. It is important to note that any given analytic focus should not diminish the relevance of other levels of analysis (Hayes et al., 2021). Thus, the PAIRS does not eschew the importance of careful analysis of behavior using information gathered around proximal antecedent and consequent variables. Instead, the PAIRS aims to expand on this information to provide practitioners with a systematic and practical way of identifying and addressing a wide range of difficulties, utilizing the level of analysis that is pertinent and helpful to a given situation.

## Strengths, Limitations, and Future Directions

Within this research, a systematic literature review of the barriers and facilitators to parent engagement was not conducted. However, as previously outlined, a growing literature base exists on this topic, and collation of common barriers and facilitators from over sixty research articles was possible without utilising a systematic review methodology. The eleven barriers and six facilitators represent a synthesis of the specific factors found in a sample of the literature, which had numerous areas of overlap. It is recognised that this is not necessarily a definitive list, and the PAIRS is designed to be a flexible and adaptive tool that can continue to be updated with new literature as well as individualized to a specific family’s unique strengths and needs.

The present study utilizes a methodology for content validation that is novel in the field of behavior analysis. The CVST was adapted from a medical context to fit the parameters of the PAIRS. By recruiting an expert panel to evaluate the content validity of each barrier-solution pairing and consulting a subset of that panel to evaluate the tool as a whole, the PAIRS is thus developed with the expertise of the professionals it is designed for. While the present study focused on developing the PAIRS based on the available literature and conducting two rounds of expert review to evaluate its content validity, it is recognised that the sample of fifteen BCBAs, and especially the smaller subset of four BCBAs who participated in the second evaluation, may not fully represent the professional experiences of behavioral practitioners working in a variety of contexts. Therefore, future research will examine the use of the PAIRS with larger samples within real-world clinical practice. The PAIRS is a tool developed by BCBAs for BCBAs and related practitioners (e.g. behavioral psychologists, assistant behavior analysts). In this way, the current intended use of the PAIRS is limited to behavioral PIIs. Given the novelty of the PAIRS, the research team wishes to avoid overextending its reach at the outset. As further research is conducted using the tool to complement evidence-based PIIs, there may be scope to adapt and expand its utility for professionals from other disciplines (e.g., early childhood educators, early interventionists) who likewise work with families of children with autism.

Finally, the present study focused on evaluation of the tool by a panel of professionals. Direct parent input was not sought at this stage of tool development for a few reasons. First, the barriers, facilitators, and solutions in the PAIRS were drawn from the literature presenting parents’ views in the form of interviews (e.g., Amsbary et al., 2020; Botterill et al., 2019; Freuler et al., 2014) or surveys (e.g., Bowker et al., 2011; DuBay et al., 2018; Rovane et al., 2020). Thus, the tool is created from the experiences and recommendations of the parents it is intended to serve, using research that is already available. Second, parent feedback would likely be most valuable and representative when the PAIRS is used in practice to address facilitators and barriers that are relevant to their unique situation. Finally, parent participation at a later stage of tool development is also likely to benefit families, as the PAIRS will have gone through the present evaluation and piloted before it is administered. Therefore, future research will focus on the family’s evaluation of the revised tool’s goals, procedures, and outcomes (i.e., social validity; Wolf, 1978) when it is used in clinical practice.

## Summary

The PAIRS was developed and revised in conjunction with an expert panel of BCBAs through two rounds of content evaluation to fill a gap in parent training literature and practice. It is a systematic, practical tool designed to complement evidence-based PIIs. Parent engagement is an essential component of PIIs, but can be impacted by numerous barriers. The PAIRS is the first tool designed to help practitioner assess barriers and facilitators to engagement, individualize their parent training approach, and respond to stressors using a contextual, functional approach.
